# Peroxisome Proliferator-Activated Receptors and Shock State

**DOI:** 10.1100/tsw.2006.298

**Published:** 2006-12-28

**Authors:** Emanuela Esposito, Salvatore Cuzzocrea, Rosaria Meli

**Affiliations:** ^1^ Department of Experimental Pharmacology, University of Naples “Federico II”, Naples, Italy; ^2^ IRCCS Centro Neurolesi “Bonino-Pulejo”, Messina, Italy; ^3^ Department of Clinical and Experimental Medicine and Pharmacology, Torre Biologica Policlinico Universitario, Messina, Italy

**Keywords:** PPARs, sepsis, nonseptic, hemorrhagic shock

## Abstract

Peroxisome proliferator-activated receptors (PPARs) are members of the nuclear hormone receptor superfamily of ligand-activated transcription factors that are related to retinoid, steroid, and thyroid hormone receptors. Three isotypes of PPARs have been identified: alpha, beta/delta, and gamma, encoded by different genes and distributed in various tissues. PPARs are implicated in the control of inflammatory responses and in energy homeostasis and, thus, can be defined as metabolic and anti-inflammatory transcription factors. They exert anti-inflammatory effects by inhibiting the induction of proinflammatory cytokines, adhesion molecules, and extracellular matrix proteins, or by stimulating the production of anti-inflammatory molecules. Moreover, PPARs modulate the proliferation, differentiation, and survival of immune cells. This review presents the current state of knowledge regarding the involvement of PPARs in the control of inflammatory response, and their potential therapeutic applications in several types of shock, as well as hemorrhagic, septic, and nonseptic shock.

## INTRODUCTION

Peroxisome proliferator-activated receptors (PPARs) were identified during the last decade and were classified as orphan members of the nuclear receptor superfamily[1,2]. These receptors, which are related to retinoid, steroid, and thyroid hormone receptors[3], bind to and are activated by fatty acids, eicosanoids, and numerous structurally dissimilar xenobiotics, known collectively as peroxisome proliferators.

PPARs are now considered as regulators of lipid and lipoprotein metabolism, possible molecular determinants of metabolic disorders, and controllers of cellular differentiation and cancer development[4]. Moreover, they play a role in inflammatory response and inflammation-related disorders[5,6]. In this article, we will focus on the new insights indicating an involvement of PPARs in several types of shock, as well as hemorrhagic, septic, and zymosan-induced shock. These findings highlight the potential of natural and synthetic PPAR agonists as therapeutic targets for the treatment of shock and related disorders.

## ISOFORMS AND STRUCTURAL FEATURES OF PPARS

To date, three subtypes of PPARs, encoded by separate genes, have been identified: PPAR-α, PPAR-γ, and PPAR-β (also called -δ)[7,8,9]. Most tissues in humans and rodents have all three receptor types, although there is considerable variability in the relative expression. PPAR-α is mostly present in tissues characterized by high rates of fatty acid catabolism, such as liver, kidney, heart, and muscle[10], whereas PPAR-γ is highly expressed in adipose tissue[11] and is also detected in mammary gland and many other tissues[12,13]. Moreover, PPARs are expressed in immunological and vascular wall cell types; PPAR-α and PPAR-γ are expressed in primary cultures of endothelial[14,15,16,17,18] and smooth muscle cells[19,20], and in monocyte/macrophages[21,22,23,24]. Whereas PPAR-α is present in isolated human monocytes and its expression increases during the differentiation process, PPAR-γ is not detectable in human monocytes, but is strongly induced on differentiation[21]. In addition, PPAR-α and PPAR-γ colocalize with specific markers of macrophages in the subendothelial region and in the lipid core of atherosclerotic lesions, smooth muscle cells, and foam cells[15,16,24,25,26]. The third member of the PPAR family, PPAR-*δ,* is expressed in a wide range of tissues including heart, adipose tissue, brain, intestine, muscle, spleen, lung, and adrenal glands[10,11].

PPARs and other class 1 nuclear receptors, i.e., retinoic acid receptor (RARs), the tyroid hormone receptors (TRs), and the steroid and xenobiotic receptors (SXR), possess similar structural and functional features. Principally, four functional domains have been identified and called A/B, C, D, and E/F. First, the N-terminal region A/B allows a ligand-independent activation domain function 1 (AF-1)[27] responsible for the phosphorylation of PPAR. Second, the C domain or the DNA binding domain (DBD) promotes the binding of PPAR to the peroxisome proliferator response element (PPRE) in the promoter region of target genes[28]. Third, the D site is a docking domain for cofactors and, at last, the E domain or ligand-binding domain (LBD) is responsible for ligand specificity and activation of PPAR binding to the PPRE, which increases the expression of targeted genes. Recruitment of PPAR cofactors to assist the gene transcription processes is carried out by the ligand-dependent activation function 2 (AF-2), which is located in the E/F domain[29].

## MECHANISM OF ACTION

Once activated by a ligand, PPARs form a heterodimer with the retinoid X receptor (RXRs), a member of the class 2 nuclear receptors that are activated by binding of 9-cis retinoic acid[30]. The interaction with the RXR allows the recruitment of a set of cofactors and the binding of the heterodimer to the PPRE in the promoter region of target genes, modulating the transcription[31,32,33]. In the absence of a ligand, to prevent PPAR/RXR binding to DNA, high-affinity complexes are formed between the inactive PPAR/RXR heterodimers and corepressor molecules, such as nuclear receptor corepressor or silencing mediator for retinoic receptors.

Binding of a ligand to the heterodimer results in the release of the corepressor from the complex, which in turn results in the binding of the activated heterodimer to the response element in the promoter region of the relevant target gene(s), resulting in either the activation or suppression of a specific gene[34].

After binding of the ligand, the recruitment of coactivator proteins, i.e., CREB-binding protein, and p-300[35] can also be required for transcriptional interaction of PPAR-γ with motifs in the PPRE. The limited availability of these coactivators might inhibit the activation of the transcription factors AP-1, NF-κB, and STAT, and consequently, inhibits the proinflammatory gene expression[36].

PPARs can also repress gene transcription by negatively interfering with the NF-κB, STAT, and AP-1 signaling pathways in a DNA-binding independent manner[15,19,21,22,37]. Negative interference by PPARs with these signal transduction pathways is likely due to a combination of protein-protein interaction and cofactor squelching, as has been previously demonstrated for other members of the nuclear receptor family, such as the retinoic acid and glucocorticoid receptors[38]. PPARs interfere with the AP-1 and NF-κB signaling pathway by preventing the binding of AP-1 and NF-κB proteins to their target sequences[14,15]. Furthermore, PPARs interact with c-*Jun*[39], as well as with p65[40].

This transrepression activity likely constitutes the mechanistic basis for the anti-inflammatory properties of PPARs.

## SYNTHETIC AND NATURAL LIGANDS OF PPARS

PPARs are activated by fatty acids and fatty acid–derived molecules, as well as by a number of pharmacological and synthetic compounds. PPAR-α, which controls genes relevant to the regulation of lipid metabolism and inflammatory process, was the first member of the PPAR family identified, and it is believed to be solely responsible for the effects of the peroxisomal proliferators[41]. To date, more than 70 compounds, including synthetic hypolipidemic fibrate drugs (Wy-14,643, nafenopin, clofibrate)[42], phthalate plasticizers (monoethylhexylphthalate), chlorinated hydrocarbons, and herbicides, along with endogenous hormones and fatty acids (arachidonic acid), have been identified as PPAR-α agonists[43,44,45].

In particular, PPAR-α is activated by naturally occuring eicosanoids derived from arachidonic acid through the lipoxygenase pathway, such as 8-S-hydroxyeicosatetraenoic acid (8-S-HETE) and leukotriene B4 (LTB4)[44,45,46], and by oxidized low-density lipoproteins (OxLDL)–derived oxidized phospholipids[47].

It has been demonstrated that PPAR-γ can be activated by arachidonic acid metabolites derived from the cycloxygenase and lipoxygenase pathways, such as 15-deoxy-*D*-12,14-prostaglandin J2 (15d-PGJ2) and 15-HETE[48,49,50]. Recent findings have revealed a crucial role of 12/15-lipoxygenase in the generation of endogenous PPAR-γ ligands[51]. In addition, fatty acid–derived components of OxLDL, such as 9-hydroxyoctadecadienoic acid (9-HODE) and 13-hydroxyoctadecadienoic acid (13-HODE), are natural ligands for PPAR-γ[50]. Finally, the antidiabetic glitazones, currently used as insulin sensitizers, are synthetic high-affinity ligands for PPAR-γ[52]. A novel series of trisubstituited isoxazoles, as PPAR-δ–selective agonists, was investigated and optimized. Furthermore, some fibrates have recently been described as specific ligands for the PPAR-δ subtype[53,54,55].

In addition to fibrates and glitazones, other pharmacological compounds have been identified as PPAR activators. Inhibition of cycloxygenase by NSAIDs (nonsteroidal anti-inflammatory drugs) constitutes a clinical approach for the treatment of inflammatory states. Lehmann et al.[56] have demonstrated that certain NSAIDs, including indomethacin and ibuprofen, are activators of PPAR-γ, acting in the micromolar range. These data are consistent with the observation that indomethacin can promote terminal adipocyte differentiation of various preadipocyte cell lines *in vitro*[57]. The molecular basis underlying this adipogenic action could thus be mediated via activation of PPAR-γ. Certain NSAIDs are also ligands for the PPAR-α form[56]. Several NSAIDs have marked effects on peroxisome activity in rodent hepatocytes when used either *in vitro* or *in vivo*[58,59], and it appears likely that these effects are mediated by PPAR-α activation.

## SHOCK STATES

Sepsis is defined as infection with evidence of systemic inflammation, consisting of two or more of the following: increased or decreased temperature or leucocyte count, tachycardia, and rapid breathing. Septic shock is sepsis with hypotension that persists after resuscitation with intravenous fluid. Normally, the immune and neuroendocrine systems tightly control the local inflammatory process to eradicate invading pathogens. When this local control mechanism fails, systemic inflammation occurs, converting the infection to sepsis, severe sepsis, or septic shock[60].

Sepsis can be caused by infection with Gram-negative bacteria, Gram-positive bacteria, fungi (and particularly *Candida*), or viruses.

Sepsis may also occur in the absence of these challenges, and in these cases, microbial toxins, particularly Gram-negative bacterial endotoxin (LPS), and endogenous cytokine production have been implicated as initiators and mediators[61,62]. Although activation of the immune system during microbial invasion is generally protective, septic shock develops in a significant number of patients as a consequence of a poorly regulated immune response to pathogens.

Despite recent progress in antibiotics and critical care therapy, sepsis is still associated with a high mortality rate (~40–50%), even when appropriate therapy is administered to the patient.

Many mechanisms are involved in the pathophysiology of septic shock, including the release of cytokines; the activation of neutrophils, monocytes, and microvascular endothelial cells; as well as the activation of neuroendocrine reflex and plasma protein cascade systems (complement system, the intrinsic and extrinsic pathways of coagulation, and the fibrinolytic system).

Several studies have shown that hemorrhagic shock results in a rapid decrease in cardiac output and organ blood flow[63]. The depressed organ perfusion and excessive production of proinflammatory mediators play an important role in the development of multiple organ dysfunction (MOD) following hemorrhagic shock[64,65]. It has been reported that systemic IL-6 levels increase following trauma-hemorrhage, and a sustained elevation in plasma IL-6 levels is correlated with the evolving organ dysfunction[66,67,68]. IL-6 has been described as a multifunctional cytokine, produced during the acute phase by macrophages, T and B cells, and nonimmune cells, such as myocytes or endothelial cells[69,70,71].

## PPARS AND SEPTIC SHOCK

Various studies have demonstrated that PPAR-γ ligands reduce the multiple organ injury/dysfunction caused by shock. In a model of Gram-negative shock (*Escherichia coli* endotoxin), pretreatment of rats with 15d-PGJ2 prevented the multiple organ failure (MOF) (i.e., hepatic and pancreatic dysfunction/injury) induced by endotoxin[72,73]. The PPAR-γ antagonist GW9662 partially reverted the beneficial effects of 15d-PGJ2, demonstrating that the mechanisms of the protective effects of 15d-PGJ2 are partly PPAR-γ dependent.

In a model of polymicrobial shock, pretreatment with 15d-PGJ2 attenuated the organ injury/dysfunction caused by coadministration of *E. coli*-LPS or *Streptococcus aureus* peptidoglycan in rats. There is evidence that both G+ and G- bacteria have been isolated in the blood of a large portion of septic patients and these polymicrobial infections often have higher mortality than infections due a single bacterium. The protection afforded by the cyclopentenone prostaglandin was reduced by GW9662[74]. Moreover, in a rat model of polymicrobial sepsis induced by cecal ligation and puncture, treatment with 15d-PGJ2 or ciglitazone ameliorated hypotension and vascular injury; reduced neutrophil infiltration in lung, colon, and liver; and reduced the elevation of cytokine in plasma. The reduction of the inflammatory process was also associated with significant improvement in animal survival. The beneficial effects of these PPAR-γ ligands were reported to be secondary to a negative modulation of NF-κB and AP-1 signal transduction pathways[75].

It is widely accepted that 15d-PGJ2 attenuates the NF-κB–mediated transcriptional activation of many proinflammatory genes by PPAR-γ–dependent and –independent mechanisms[76]. For instance, 15d-PGJ2 attenuates the formation of the cytokines, such as tumor necrosis factor-α (TNF-α) and IL-12[77], the expression of vascular cell adhesion molecule-1 (VCAM-1) and intercellular adhesion molecule-1 (ICAM-1)[78], and the expression of the inducible proinflammatory proteins, COX-2, cytosolic phospholipase A2[79], and inducible nitric oxide synthase (iNOS)[22,80]. There is, however, also evidence that 15d-PGJ2 may enhance the formation of the proinflammatory chemokine IL-8 in human macrophages/monocytes stimulated with endotoxin in a PPAR-γ–dependent fashion[81].

However, the role of PPAR-γ activation and the precise molecular mechanisms of 15d-PGJ2 have not been fully determined in sepsis. Administration of 15d-PGJ2 improved survival, and reduced lung injury and neutrophil trafficking to lung and small intestine in mice subjected to endotoxic shock[82]. 15d-PGJ2 enhanced the PPAR-γ function in the lung, decreased NF-κB activity, and enhanced the expression of the cytoprotective protein HSP70. The heat shock response is a highly conserved cellular defense mechanism to injury, which is characterized by the increased expression of chaperone proteins that provide cytoprotection from inflammatory insults, including oxidative stress, viral infection, and ischemia-reperfusion injury[83,84]. 15d-PGJ2 and activation of PPAR-γ led to enhancement of the heat shock response in the lung as demonstrated by the increased expression of HSP70, which correlated well with improvement of lung injury. These findings are in agreement with previous *in vitro* reports demonstrating that 15d-PGJ2 may induce expression of HSPs. Maggi and colleagues have proposed that the ability of 15d-PGJ2 to block cytokine-induced iNOS in macrophages is associated with the expression of HSP70[85].

Moreover, the therapeutic effect of 15d-PGJ2 may be consequent to prevention of gene expression of molecules controlling neutrophil rolling, firm adhesion, and transmigration. *In vitro*, PPAR-γ activation suppressed the VCAM and the ensuing leukocyte recruitment in endothelial cells[86,87,88]. Similarly, *in vivo* treatment with PPAR-γ ligands suppress neutrophil recruitment in major organs in rats with polymicrobial sepsis[75]. Several cellular mechanisms, including the mode of gene regulation and signal transduction, may account for the anti-inflammatory properties of 15d-PGJ2 in endotoxic shock. Activation of NF-κB, a critical factor in the coordination of both innate and adaptive immune responses in sepsis, is rapid and occurs within minutes after the microbial challenge[89,90]. The duration and the degree of NF-κB activity appear to correlate with the severity of the inflammatory response and to persist longer in nonsurviving than surviving patients with acute sepsis[91]. It has been proposed, in fact, that PPAR-γ may modulate expression of inflammatory genes by direct transrepression. PPAR-γ inhibited expression of the iNOS by direct interaction with CREB-binding protein, thus limiting its availability for NF-κB and AP-1 transcription[36,92].

The effect of PPAR-γ ligands on the inflammatory response linked to shock appears to be complex and dependent on the specific PPAR-γ ligands used, corepressors or coactivators involved, the mode of immune cell activation, and the mediators that are measured.

More recently, hearts from mice treated with LPS were examined for altered expression of PPAR-γ and coactivators[93]. LPS-treated mice exhibited reduced expression of all isoforms of PPAR-γ and RXRa and thyroid receptor (TR). Additionally, the coactivators CBP/P300, steroid coactivator (SRC-1) and SRC-3, TR-associated protein 220, and PPAR-γ coactivator (PGG-1) were reduced. Because the nuclear receptors, cofactors, and the genes that they regulate are important in myocardial fatty acid oxidation, reduced expression of these factors may contribute to altered myocardial fatty acid metabolism and contractility in endotoxic shock[93]. The cardiovascular hypodynamic phase of septic shock is associated with down-regulation of PPAR-γ expression on the endothelium of thoracic aortas and in the bronchial epithelium in rats[75]. Conversely, it has been demonstrated[94] that in porcine white blood cells, PPAR-γ protein expression increased twofold over basal in response to acute endotoxemia, suggesting a different regulation of PPAR-γ expression in various cell types.

Septic shock is often associated with vascular damage, hemostasis activation, and development of disseminated intravascular coagulation. In fact, in human volunteers, endotoxin injection is related to prolonged coagulation activation and endothelial injury[95]. It has been demonstrated that during septic shock, an alteration of nitric oxide pathway occurs. The reduction of endothelial constitutive NOS (eNOS) expression and overexpression of vascular smooth muscle cell iNOS contribute to hypotension and observed alteration of tissue perfusion[96,97,98].

On the basis of anti-inflammatory effects of PPAR-α agonists and their prevention of coagulation activation, very recent studies suggest a possible role for PPAR-α in the modulation of sepsis-induced tissue and vascular injury.

Fenofibrate, a well-known PPAR-α agonist, has been shown to inhibit up-regulation of monocyte tissue factor (TF) expression in monocytes and macrophages, negatively interfering with the AP-1 and/or NF-κB pathway[99]. Moreover, this synthetic drug inhibits transcription of cytokine-induced VCAM-1 gene, preventing leukocyte adhesion and associated endothelial injury, at least in part via inhibition of the NF-κB pathway[15,100].

Recently, for the first time, evidence was provided that PPAR-α activation has a protective effect in septic shock, using a well-characterized rabbit endotoxin-induced shock model[101]. This effect was associated with an improvement in endothelial relaxation function and a decrease in coagulation activation (monocyte TF expression).

As previously reported[102,103], LPS induces a decreased sensitivity to phenylephrine (PE) that is improved by L-NAME (Nω-nitro-L-arginine methyl ester). This suggests the presence of iNOS in smooth muscle cells induced by LPS. Moreover, removal or damage of endothelial cells can also trigger the induction of iNOS in vascular smooth muscle cells[104]. In rabbit shock model, the LPS-induced decrease in sensitivity to PE in smooth muscle cell was restored by fenofibrate treatment, suggesting reduction in iNOS

expression[101]. The mechanism underlying the decrease in iNOS expression in smooth muscle cell, after fenofibrate treatment, is unknown, but may be due at least in part to restoration of endothelial structure and function induced by PPAR-α activation or direct effect of PPAR-α activation.

Several data show that LPS is responsible for abnormal coupling between acetylcholine (Ach) endothelial receptor and eNOS. Oxidative stress has been incriminated in this uncoupling[105,106,107] and PPAR-α activation prevents this effect, increasing antioxidant enzyme release, i.e., catalase and superoxide dismutase[108,109].

Few studies examined the role of PPAR-δ in the inflammatory responses. Ding et al.[110] investigated the effects of PPAR-δ and its synthetic ligand GW0742 on TNF-α production in cultured cardiomyocytes. This study indicated that a PPAR-δ–selective ligand inhibited LPS-induced TNF-α production from cardiomyocytes. Moreover, activation of PPAR-δ abrogated LPS-induced degradation of IκBs, thus suppressing LPS-induced NF-κB activities. Therefore, PPAR-δ could be an important determinant of TNF-α expression via the NF-κB signaling pathway, thus serving as therapeutic targets to attenuate inflammation.

Moereover, Letavernier et al.[111] recently showed that wild-type mice that were given the specific PPAR-δ ligand L-165041 before renal ischemia were completely protected against renal dysfunction. This protective effect was accompanied by a significant reduction in medullary necrosis, apoptosis, and inflammation. On the basis of *in vitro* studies, PPAR-δ ligands seem to exert their role by activating the antiapoptotic Akt signaling pathway. These results pointed to PPAR-δ as a remarkable new target for preconditioning strategies.

## PPARS AND NONSEPTIC SHOCK

Zymosan-induced shock is an animal model used for improving and understanding the pathophysiological mechanism of MOF. Zymosan, a nonbacterial, nonendotoxic agent, produces acute peritonitis and MOF characterized by functional and structural changes in liver, intestine, lung, and kidneys[112,113,114].

Recent studies have shown that macrophages play an important role in zymosan-induced inflammatory processes by activating the synthesis of cytokines and proinflammatory mediators[114,115]. Zymosan administration in the peritoneal cavity is associated with systemic hypotension, high peritoneal and plasma levels of nitric oxide, maximal cellular infiltration, exudate formation, and cyclooxygenase activity[116].

Recently, treatment of mice with rosiglitazone (1 and 6 h after zymosan) attenuated the peritoneal exudation and the migration of polymorphonuclear (PMN) cells. Rosiglitazone also reduced the lung, liver, and pancreatic injury and renal dysfunction caused by zymosan as well as the increase in myeloperoxidase (MPO) activity and malondialdehyde concentrations in the lung and intestine. Immunohistochemical analysis for iNOS, nitrotyrosine, and poly(adenosine diphosphate-ribose) revealed positive staining in lung and intestine tissues obtained from zymosan-treated mice. The degree of staining for these markers was clearly reduced in tissue sections obtained from zymosan-treated mice that received rosiglitazone. The protective effects of rosiglitazone was related to activation of the PPAR- γ receptor, since a PPAR-γ antagonist, GW 9662, abolished the protective effect of rosiglitazone[117].

Moreover, Marzocco et al.[118] investigated the effect of 15d-PGJ2 on this nonseptic shock model in mice. This study provides the first evidence that pretreatment of mice with 15d-PGJ2 attenuates the development of induced peritonitis, reducing the PMNs infiltration and tissue damage of the intestine, as assessed by MPO activity and histological examination, respectively. Moreover, the cyclopentenone prostaglandin reduces NO both in the peritoneal exudates and plasma. The reduction of NO levels by 15d-PGJ2 may contribute to the attenuation of the formation of nitrotyrosine in the ileum from zymosan-treated mice.

These findings support the potential use of PPAR-γ ligands as therapeutic agents in the therapy of conditions associated with nonseptic shock.

## PPARS AND HEMORRHAGIC SHOCK

Hemorrhagic shock is a condition characterized by rapid and significant loss of intravascular volume, which may lead sequentially to hemodynamic instability, decreases in oxygen delivery, decreased tissue perfusion, cellular hypoxia, organ damage, and can be rapidly fatal.

Severe hemorrhage impairs the delivery of oxygen and nutrients to the tissues, and produces a state of shock that induces a cascade of proinflammatory cytokines that are associated with immunosuppression, hemodynamic depression, and organ dysfunction[119,120]. The therapeutic goals for hemorrhagic shock are to stop bleeding and to restore intravascular volume[121].

Abdelrahman and colleagues[122] recently investigated the role of PPAR-γ in organ injury associated with hemorrhagic shock. In particular, the authors showed that 15d-PGJ2 attenuated the renal, hepatic, lung, and intestinal injury/dysfunction associated with hemorrhage and resuscitation. In contrast, 15d-PGJ2 did not affect the circulatory failure (delayed fall in mean arterial blood pressure) associated with hemorrhagic shock. However, a pretreatment regimen was used and further studies employing a therapeutic (post-treatment) administration of 15d-PGJ2 are warranted.

In this model, GW9662 not only reversed the protective effects afforded by 15d-PGJ2, but also augmented the degree of liver injury caused by hemorrhage and resuscitation[122]. This finding, therefore, indicates that hemorrhage and resuscitation results in the release of endogenous PPAR-γ ligands and that the amounts of these ligands released are sufficient to protect the liver against the organ injury associated with hemorrhagic shock, but not septic shock[123].

## CONCLUSIONS

Many mechanisms are involved in the pathophysiology of shock state, including the release of cytokines; the activation of neutrophils, monocytes, and microvascular endothelial cells; as well as the activation of neuroendocrine reflex and plasma protein cascade systems (Fig. 1). There is extensive complementary and synergistic interaction of the different components in the progression of shock to MOD and/or MOF. Timely, aggressive resuscitation to preserve organ function remains a fundamental principle in the care of shock. The specific treatment is directed at identifying and treating the underlying disorder.

PPARs, known at the beginning as regulators of lipid and lipoprotein metabolism, more recently have been identified as important players in the metabolism of lipid-derived inflammatory mediators and in inflammation-related pathologies. The reported observations and new knowledge about the role of PPAR agonists on inflammatory response[124,125], and epithelial dysfunction indicate that they could be effective in the treatment of shock and related disorders.

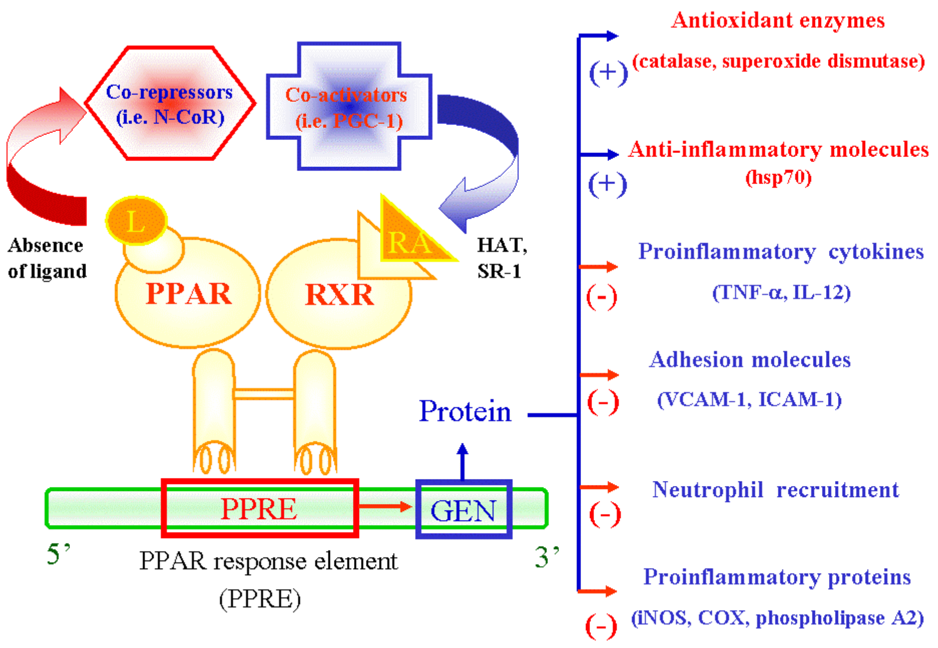

**FIGURE 1.** Similar to other nuclear hormone receptors, PPAR acts as a ligand-activated transcription factor. PPAR-α, when activated after binding with specific ligand, interacts with RXR and regulates the expression of target genes. These genes are also involved in the catabolism of fatty acids. Conversely, PPARs are activated by different ligands and affect the expression of genes involved in the storage of fatty acids. PPAR-β is only weakly activated by fatty acids, prostaglandins, and leukotrienes, and has no known physiologically relevant ligand.
